# Dissemination and implementation of a policy on school health in public schools: A systematic review

**DOI:** 10.4102/curationis.v43i1.2110

**Published:** 2020-12-10

**Authors:** Helen I. Wankasi, Leepile A. Sehularo, Mahlasela A. Rakhudu

**Affiliations:** 1 School of Nursing Science, Faculty of Health Sciences, North-West University, Mmabatho, South Africa

**Keywords:** dissemination, implementation, school health policy, public schools, policy and systematic review

## Abstract

**Background:**

The need to achieve school health and promote well-being that would transcend children’s school life has been highlighted in several studies. Promotion of health and well-being of children has not been achieved despite the prescripts of the World Health Organization and national mandates.

**Objectives:**

The purpose of this systematic review was to explore and describe the current evidence on the dissemination and implementation of a policy on school health in public schools.

**Methods:**

Five steps of a systematic review were used to achieve the purpose of the study. The steps include framing a clear review question, developing a search approach through gathering and classifying evidence, conducting a critical appraisal, evidence summary as well as the results. Ebscohost, SAE publications, Web of Science and JSTOR databases were used to identify articles written between 2013 and 2018 and to enable access to current studies on the promotion of school health. Keywords included the following: dissemination; implementation; school health policy; and public schools. The search yielded *n* = 1995 articles. From this figure, 1976 articles were ineligible and only 19 articles met the inclusion criteria.

**Results:**

Seven themes emerged from the findings of this systematic review as follows: shared information, training and development of key role-players, programme development and research, commitment from key role-players, monitoring activities, executive support and collaborative partnerships.

**Conclusion:**

The findings show that it is possible for a policy on school health to be disseminated and implemented effectively in public schools.

## Introduction

Many countries such as United States of America, Australia, Somalia and South Africa have school health services covering a population of about 1.3 billion children in public elementary and secondary schools (Baltag, Pachyna & Hall 2015:270; Kolbe 2019:443). Yet, the dissemination and implementation of a policy on school health are still ineffective in many countries (Mohlabi, Van Aswegen & Mokoena 2010:250; Saito et al. 2014:846). The ineffective implementation of such policy has created negative consequences on school children, such as sicknesses and absenteeism (O’Dea 2012:78; Ofovwe & Ofili 2010:1578). Migraine is common and underdiagnosed amongst secondary school students in Benin City, Nigeria, and negatively impacts on the quality of life including school absenteeism (Ofvwe & Olifi 2010:1578). In all of these studies, the aims, objectives, method and population were contrary to the systematic review conducted in this study primarily because the focus of each of these studies was to highlight and provide evidence based on a particular school health service and not on the policy in its entirety. This systematic review focusses on current evidence on the dissemination and implementation of a policy on school health. Exploration and description of current evidence on a policy on school health will assist practitioners involved in school health services (such as teachers, healthcare professionals, school health policy makers, children and future researchers) to have a comprehensive directive with regard to the dissemination and implementation of a policy on school health.

In Europe, Lee and Gortmaker (2012:429) described current dissemination and implementation evidence as contemporary scientific evidence, which supports comments of mutual interest offered with regard to the dissemination and implementation of school health services. Although Lee and Gortmaker (2012:425) extensively described the need for effective dissemination and implementation to achieve the objectives of the public health system in improving population health with special focus on schools, these authors equally emphasised the need to reduce health inequalities to a very low ebb and promote enduring educational prospects, but no attention was paid to the dissemination and implementation of a policy on school health.

From the perspective advanced by Lee and Gortmaker (2012:428), it could be inferred that mutual interest is crucial in easily building a relationship amongst stakeholders involved in school health sequel to underpinnings such as what all relevant stakeholders stand to benefit and share principles with regard to sustainable and effective dissemination and implementation to promote the health of school children. These stakeholders include the teachers, school health nurses and educational psychologists. Thus, if the literature has consistently claimed there is poor dissemination and inappropriate implementation of a policy on school health (Mohlabi et al. 2010:250; Saito et al. 2014:846), then there is a need to explore and describe current evidence with regard to the dissemination and implementation of a policy on school health for stakeholders to have an idea of current and operational evidence. Such need is on the premise that a policy on school health is a document developed as a framework for decision-making and rules to guide stakeholders in the implementation of all other services related to school health.

In Australia, such evidence could be found in the implementation and improvement of food items sold in school canteens (Delaney et al. 2017:1315; Wolfenden et al. 2017:6). The idea was to curb school children’s indulgence in unhealthy food habits from accessible food items in canteens. In other words, in these studies, the authors focussed on school feeding and menu issues, which are only programmes enshrined in school health policies to ensure school children eat healthily and not its effective dissemination for stakeholders to be informed and implement it thereof for school children to study with maximum concentration.

In Africa, a number of studies have been conducted on school-based health promotion issues (Moodley et al. 2013:320). These authors established the successful implementation of human papilloma virus (HPV) vaccination amongst female school children between the ages of 9 and 12 in collaboration with the school health team in KwaZulu Natal. The inevitability of the use of existing school health teams in achieving the aim of a project was established in the study. Thus, there is need for further exploration and description with regard to the selection of current dissemination and implementation evidence.

The above information would enable stakeholders to discover, understand and promote validated assessment of current existing evidence for the dissemination and implementation practices whilst maintaining global school health promotion standards in different contexts. Significantly, this systematic review would be the first review conducted on evidence that would be a reference point in health and educational practices alongside the field of research in terms of dissemination and implementation of a policy on school health.

## Methodology

A systematic review was followed to achieve the aim of the study. A five-step systematic review pinpoints clues were used to gather and summarise the best available research evidence to enhance decision-making and promote the use of evidence recommended (University of York, Centre for Reviews and Dissemination [CRD] 2017:72). Preferred reporting items systematic and meta-analysis (PRISMA) flow chart was used to document evidence of the studies included in this systematic review (University of York, Centre for Reviews and Dissemination 2017:82). The following steps were followed in this systematic review.

### Step 1: Framing a clear review question

Framing a clear review question was the first step in this systematic review. The Participants, Interest, Context, Study design (PICOS) framework was used in this study to identify elements of a clearly focussed systematic review question as shown in Table 1 (University of York, CRD 2017:72).

**TABLE 1:** Participants, Interest, Context, Study design with elements of the review question.

Code	Description
P	Participants of interests
I	Interest was on evidence for the dissemination and implementation of policies on school health
CO	Context (this is a context or setting and the distinct features are health policies of public schools)
S	This refers to the adopted study design

*Source*: University of York, Centre for Reviews and Dissemination, 2017, *Systematic reviews: CRD’s guidance for undertaking reviews in health care*, p. 72, University of York, Centre for Reviews & Dissemination, University of New York, New York.

The systematic review question was as follows: ‘What is the evidence available with regard to the dissemination and implementation of a policy on school health in public elementary and secondary schools?’.

### Step 2: Developing a search approach through gathering and classifying evidence

This second step of the systematic review involved gathering and classifying evidence through a well-planned process to showcase the search protocol, minimise bias and highlight the inclusion and exclusion criteria to document best articles that provided evidence to answer the framed systematic review question.

#### Search procedure

This section focusses on the search protocol adopted in the study such as evidence acquisition, inclusion and exclusion criteria, study selection, data extraction and quality assessment and synthesis (University of York, CRD 2017:79).

#### Gathering and classifying evidence

To conduct an effective search, ambiguity and bias were avoided, and the researcher consulted two librarians, who assisted in creating a search procedure from relevant databases that had a bearing on the subject under investigation. The search period covered articles posted from 1st January 2013 to 31 December 2018, linking key terms such as ‘dissemination, implementation, programmes, services and policy on school health’ to enable access to relevant studies in the field. The rationale for focussing on studies from 2013 to 2018 was to enable access to current studies on the promotion of school health. A rigorous search was conducted on selected sites from the A-Z library information services publication finders’ databases, employing separate procedures for each database and keyword areas to validate the search. For instance, Ebscohost, Web of Science, SAE publications and JSTOR databases were used to search for relevant studies by using the following inclusion and exclusion criteria.

#### Inclusion and exclusion criteria

Articles considered for the review were those that were peer-reviewed and focussed on school health policies, programmes or services and which were directly related to the subject matter. The articles were written in English and published between 2013 and 2018. Quantitative, qualitative and mixed methods were used in the study to produce more reliable findings. Both the researcher and an independent reviewer used the John Hopkins’ Nursing Appraisal Checklist (JHNAC) to determine the inclusion and exclusion of relevant studies. Duplicates were excluded.

#### Evidence documentation

The researcher and an independent reviewer commenced with the main search, keeping in mind the set criteria for relevance through the PRISMA reporting system (non-randomised controlled trials) (University of York, CRD 2017:79). The use of PRISMA allowed for consistency and provided a summary of published results.

**FIGURE 1:**
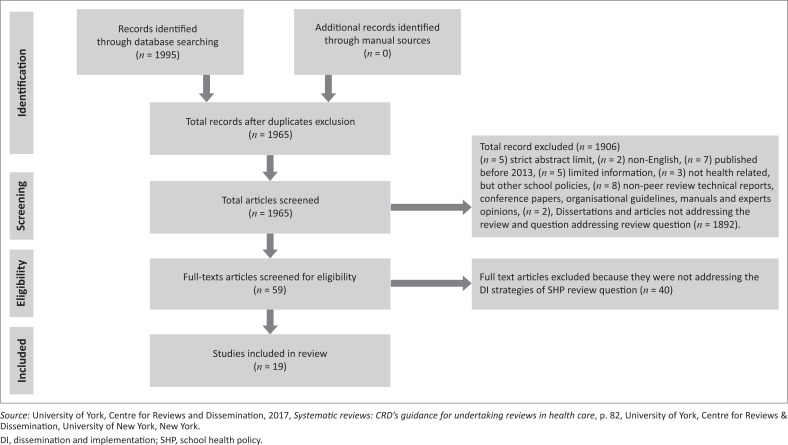
Preferred reporting items systematic and meta-analysis flow diagram of search results.

Figure 1 shows that the total number of records identified from electronic databases was 1995. No records were found through the manual search. Total records remaining after excluding 30 duplicates was 1965. Full-text articles screened for eligibility were 59, 40 of the studies were excluded because they were not addressing the school health policies and the studies included in this systematic review are 19.

### Step 3: Conducting a critical appraisal

An appraisal is a progressive examination to establish quality, successes or failures (Samuel et al. 2016:635; Webel et al. 2010:250). An appraisal was conducted with the help of John Hopkins Nursing Evidence Based Practice Appraisal (JHNEBP) tool consisting of an appraisal of all nursing research methods (Newhouse et al. 2007:132). There are three quality ratings based on quality appraisal A, B and C. A deals with high quality studies, which have consistent, generalisable results with sufficient sample size for the design of the study. Definitive conclusions and consistent recommendations of the study are based on comprehensive literature review that includes thorough reference to scientific evidence. B deals with good quality studies, which have reasonably consistent results and a sufficient sample size for the study design. There are some control and fairly definitive conclusions. Reasonably consistent recommendations of the study are based on a fairly comprehensive literature review that includes some reference to scientific evidence. C deals with studies, which have low quality or major flaws. There is little evidence with inconsistent findings. There is also insufficient sample size for the design of the study and conclusions cannot be drawn. In this review, the researcher and independent reviewer critically appraised all the relevant studies (19).

### Step 4: Evidence summary

In the fourth step, the researcher used Table 2 to assess applicability, quality of research-design, sample and rigour, findings and discussion of articles that met the eligibility criteria in JHNAC.

**TABLE 2:** Evidence table of studies included for appraisal.

Author	Design/sample	Findings	Rigour
Vanneste et al. (2016)	Semi-structured interviews Sample size: *n* = 17	It was found that collaboration, communication and monitoring; referral, dialogue and consultation as evidence to disseminate and implement a policy on school health.	Qualitative research. Appropriate population of interest. Data collection and analysis methods are indicated. Findings are presented. Conclusions are drawn from findings. Limitations are stated. Study is approved by the Medical Ethics Committee of the University Hospital Maastricht and Maastricht University. Instrument used: JHNEBP. Strength of evidence = III. Quality of evidence = B (good quality).
Larsen et al. (2017)	Quasi-experimental design Sample size: *n* = 414 students *n* = 264 parents	The RE-AIM framework was adopted in the study as evidence to assess the dissemination and implementation of nutrition knowledge in kindergarten schools.	Quantitative study and population of interest were asked questions appropriate to this review. Data collection and analysis methods are shown. Findings supported the literature and conclusions established on findings. Strengths, limitations and implications are clearly stated. Study is approved by the Institutional Review Board at Independent Review Consulting Inc. Review no. 13101-01. Strength of evidence = III. Quality of evidence = B (good quality).
Larson et al. (2017)	Cross-sectional study Sample size: *n* = 141 from *n* = 42 schools	The study revealed that information, price inclination towards nutritious food items, impromptu visits to school cafeteria, prohibition of banned marketing on food items, physical exercise, leadership and programme support are examples of evidence used to disseminate and implement a policy on school health.	Study is quantitative and population is appropriate to the interest population of the review. question. Aim and objectives are clearly stated, data collection and analysis methods presented, (descriptive statistics, equations and generalised linear regression models). Findings supported the literature and discussion. Limitations are stated and conclusions are based on findings. Scope of the study is approved by the University of Minnesota’s Institutional Review Board. Instrument used: JHNEBP. Strength of evidence = III. Quality of evidence = B (good quality).
Tapia et al. (2017)	Randomised controlled trial *n* = 746 from 18 middle schools	Utilisation of existing school programmes, adherence, sustainability, training, support, supervision, skills development, engagement, recruitment, training and supervision. Use of local resources, coordination and advocacy. Community-based stakeholder collaboration, partnership and parent–centred or family-centred approach (familiasunidas). Communication and programme manuals.	Mixed methods approach is appropriate for the study. Data collection methods are stated. Study population is appropriate with review question population of interest, and sample size is appropriate for the population. Inclusion and exclusion criteria are stated. Search protocol is clearly shown. Data analysis method is a multilevel regression equation by using MLwiN v2.26. Findings are well presented. Findings support literature and conclusion based on findings. Approval to conduct study is from the Joint Chinese University of Hong Kong – New Territories East Cluster Clinical Research Ethics Committee (Ref: 2014.114). Instrument used: JHNEBP. Strength of evidence = I. Quality of evidence = A (high quality).
Pucher et al. (2017)	Longitudinal design Pretest *n* = 90 Post-test *n* = 69 post	The study revealed sustainable collaboration, organisational knowledge or awareness, external and internal support and use of theory, shared responsibility and communication as evidences to disseminate and implement a policy on school health.	Study is quantitative and the purpose, methods of data collection and mediation analysis are all stated in the study. Findings are presented in clear terms. Findings, support literature and limitations are stated. Conclusions are drawn from the findings of the study. Department of Health Promotion, Maastricht University, Maastricht, and The Netherlands Institute of General Practice and Family Medicine, Herdecke University, Germany, approved the study. Instrument used: JHNEBP. Strength of evidence = III. Quality of evidence = A (high quality).
Weatherson et al. (2017)	Mixed method surveys, interviews and scoping systematic review Sample size: *n* = 15	Two evidences are established. Prescriptive approach; involves students. Non-prescriptive approach involves provision of opportunities, resources and facilities.	Quantitative survey and qualitative approach to inquiry. Objectives, inclusion, exclusion criteria and search protocol are clearly demonstrated with a PRISMA flow chart. Discussions and recommendations are based on findings. Strengths and limitations are appropriately stated. Study is approved by the School of Health and Exercise Sciences, Faculty of Health and Social Development University of British Columbia, Okanagan. Instrument used: JHNEBP. Strength of evidence = I. Quality of evidence = A (high quality).
Pagnotta et al. (2016)	Retrospective case study design, document analysis Sample size: *n* = 11 after obtaining data saturation	The authors found teamwork, open communication, shared responsibility, empowerment, job description, collaboration and use of experts and resource persons as evidences.	Study is qualitative and objectives of the study are stated, qualitative data collection through phones (recorded digitally and transcribed verbatim). Data analysis approach is explained. Discussion, conclusions, limitations and recommendations for future studies are based on findings. Study is approved by the Department of Kinesiology, Temple University, Philadelphia. Instrument used: JHNEBP. Strength of evidence = I. Quality of evidence = A (high quality).
Hager et al. (2016)	Online survey Sample size: school survey *n* = 744, school survey = 24	System support for school-level partnership and collaboration team. Effective communication and provision of resources.	Quantitative study. Methods of data collection and analysis were stated. Findings were stated and recommendations were based on extensive literature review and the findings. Study was approved by the University and State Health Department Institutional Review Boards in Baltimore, USA. Instrument used: JHNEBP. Strength of evidence = level II. Quality of evidence = A (high quality).
Leow et al. (2014)	Case study Sample size: *n* = 54	Collaboration, empowerment or capacity building, encouragement of teachers, committed policy implementation. Provision of grants and in-sort donations. Accountability, leadership, community partnership and staff support, advocacy and political will. Prescription of assignment and persistency.	Qualitative study. 2^o^ data evaluation 2005–2008. Qualitative study protocol is observed. Objective and methodology are stated. Interviews are conducted to generate data. Analysis is purely thematic. Results are clearly presented. Discussion is based on results. Study is approved by the School of Human Movement Studies, University of Queensland, and Australia Research Ethics Committee. Instrument used: JHNEBP. Strength of evidence = III. Quality of evidence = B (good quality).
Nathan et al. (2016)	Randomised controlled trial (RCT) Sample size: *n* = 55 schools	Multi-strategic intervention, executive support, adjusted training of stakeholders, provision of resources and tools, monitoring, evaluation, telephone calls and feedback, motivation in the form of recognition of performing schools, with continuous support.	Quantitative study. Objectives of the study were clearly stated, data collection, sample size and analysis methods (descriptive statistics) were appropriate for the study population (315) and context. Presentation of findings is clear. Conclusion was drawn from the findings and limitations were also discussed. Study was approved by the Hunter New England Human Research Ethics Committee, University of Newcastle and New South Wales, Department of Education. Instrument used: JHNEBP. Strength of evidence level = I. Quality of evidence = A (high quality).
Flannery et al. (2013)	Longitudinal study Sample size: *n* = 8 participating schools	Capacity building through professional development, evidence-based information as key source of decision-making, technical assistance with involvement of beneficiaries, development of action as a guide, and team formation. Collaboration by stakeholders, consensus building and effective communication.	Quantitative study. The purpose of the study is to evaluate a high-school-wide evaluation tool (HS-SET), a 3-point scale served as the main instrument for data collection. Data analysis methods (descriptive statistics) are stated. Results are presented clearly. Discussions and recommendations are based on literature and study findings. Limitations and the need for further research are stated. Study is approved by the University of North Carolina and supported by the Institute of Education Sciences, U.S. Department of Education. Instrument used: JHNEBP. Strength of evidence = I. Quality of evidence = A (high quality).
Chiang, Meagher and Slade (2015)	Sample sizes: *n* = 4 schools’ legislation education and health data bases, databases of two districts and four informal interviews	Collaboration through policy alignment, awareness creation, motivation, partnership, leadership and institutional support, development of an advisory committee and follow-up opportunities. Resources and stakeholder involvement or engagement. Formation of teams.	Qualitative study. The objective and data collection method (database search and informal individual interviews) are stated, and the findings are presented and discussed. Implication of framework to school health is stated. Study is published by the American School Health Association. Human subjects are not involved to warrant approval. Instrument used: JHNEBP. Strength of evidence = IV. Quality of evidence level = B (good quality).
Yoong et al. (2016)	Randomised controlled trial Sample size: *n* = 72 (36 interventions, 36 control groups)	Menu feedback reports, multi-component menu audits, telephone feedback, training, compliance. Training, financial support, theories or evidence-based sources.	Study is quantitative and the aim and objectives of study are stated. Sample size is adequate. Data collection is performed through Computer Assisted Telephone (CAT), individual interviews and single-blinded randomised controlled trial. Data are analysed by using SAS 9.3 (SAS Institute Inc., Cary, NC) by an independent statistician. Findings are discussed extensively based on literature. Limitations relating to the study are stated. Conclusions and recommendations made are also appropriate. Study is approved by the Australian New Zealand Clinical Trials Registry (ACTRN12613000543785). Instrument used: JHNEBP. Strength of evidence level = I. Quality of evidence = A (high quality).
Montague et al. (2014)	Sample size: *n* = 91–892 with an average of 317 per school from 42 high schools.	Community consultation and engagement, demand-creation, services decentralisation, awareness creation, monitoring, review and adaption are evidences to disseminate and implement a policy on school health.	Quantitative study. Multi-phased voluntary male medical circumcision (VMMC) demand creation approach adopted. Discussion, conclusions and recommendations for further studies are made based on the findings. Study is approved by the Biomedical Research Ethics Committee of the University of KwaZulu-Natal. Instrument used: JHNEBP. Strength of evidence = IV. Quality of evidence = B (good quality).
Ha et al. (2018)	Cluster randomised controlled trial Sample size: *n* = 773 from 26 schools and focus group interviews	SELF-FIT intervention consisting of teachers training, fitness dice activity, fairness, motivation, self-assessment questionnaire. Supportive teachers’ behaviour, fitness infusion, fidelity and stakeholder engagement. Integration essential for dissemination and implementation strategies.	Qualitative study. Research questions are based on PICO. The objectives, inclusion and exclusion criteria are stated. Search evidence table is shown and results are discussed. Limitations and conclusions are discussed in line with literature and findings and the need for further research is also stated. Study is approved by the Joint Chinese University of Hong Kong – New Territories East Cluster Clinical Research Ethics Committee (Ref: 2014.114). Instrument used: JHNEBP. Strength of evidence = I. Quality of evidence = B (good quality).
Robinson et al. (2014)	Observational cross-sectional study Sample size: *n* = (341 female pupils and 342 male pupils) from five elementary schools	Study revealed the mandated PE policy formulation: engage children in 30 min daily PE programmes 5 days out of the 7 days in a week by an employed trained teacher. Step count: 12 000 steps per day as evidence to disseminate and implement a policy on school health.	Quantitative study. The objectives, inclusion and exclusion criteria and data collection method are clearly stated and results are presented. Conclusion is drawn from literature and findings; limitations are also stated. University of Miami Miller School of Medicine, Miami, FL, USA. Instrument used: JHNEBP. Strength of evidence = II. Quality of evidence = B (good quality).
Reilly et al. (2016)	A randomised controlled trial Sample size: *n* = 70 schools	Policy development, compliance, monitoring and evaluation identified in the study. Direct observations and on-site visits, self-report, menu-audit as evidence.	Quantitative study. Quasi-experimental design. The ppurpose of study and data collection methods are stated. Descriptive statistics of means and standard deviations are calculated by using IBM SPSS software. Findings are clearly presented, conclusion is drawn from results and the literature is reviewed. Limitation of study, recommendations and implications of study are stated. Approval to conduct the study is given by the Institutional Review Board of Auburn University. Instrument used: JHNEBP. Strength of evidence = I. Quality of evidence = A (high quality).
Wolfenden et al. (2017)	A randomised controlled trial Sample size: *n* = 70 schools	The study revealed feedback, academic detailing, provision of financial, human and material resources, capacity building, motivation and promotion of plausible ideas as means of disseminating and implementing a policy on school health. Unanimity and leadership support.	Quantitative study. The objective and purpose of study are stated. Data collection method, appropriate sample size and analysis method are also stated. Descriptive statistics are analysed by using SAS Version 9.3. Findings are presented and discussed in consonance with literature. Recommendations are made, and limitations and strengths of the study are stated. Study is approved by the Hunter New England Area Health Service Human Research Ethics Committee and the New South Wales Department of Education, Australia. Instrument used: JHNEBP. Strength of evidence = I. Quality of evidence = A (high quality).
Nguyen et al. (2017)	Individual interviews Sample size: *n* = 17	Knowledge-building through awareness, provision of resources and regular education in the areas of nutrition in the Republic of South Africa, elementary schools identified as evidences to disseminate and implement a policy on school health.	Qualitative study. The purpose and objectives of the study are indicated. Semi-structured individual interviews are used as data collection instrument. Sample size is appropriate in consonance with the design. Data analysis method (thematic data analysis) is stated. Findings are presented, and discussion and conclusions are drawn based on the findings. The study is approved by the Non-Communicable and Communicable Diseases Research Unit, South African Medical Research Council, School of Child and Adolescent Health, University of Cape-Town, Cape-Town, Republic of South Africa. Instrument used: JHNEBP. Strength of evidence = III. Quality of evidence = B (good quality).

JHNEBP, John Hopkins Nursing Evidence Based Practice Appraisal.

#### Data extraction

Data were extracted to identify and ascertain studies that responded appropriately to the review question. A trial run data extraction was performed in the first three articles by both the first author and an independent reviewer to assess qualities and to identify current evidence. The independent reviewer, who has a PhD in Nursing, was given the three articles only when she agreed to participate in the writing process. Engaging the independent reviewer ensured reliability and coherent methodological processes and added applicable expertise in the appraisal process, considering the fact that the reviewer applied the same method in her PhD studies and had supervised postgraduate students in the field. Although there were discrepancies, these were amicably resolved by both parties.

#### Data analysis

Data analysis involved assembling the data together by adopting a narrative style (University of York, CRD 2017:83). Data analysis was conducted independently by the first author and an independent reviewer. Engaging the independent reviewer ensured reliability and coherent methodological processes and added applicable expertise in the appraisal process. In this systematic review, both the researcher and an independent reviewer engaged in a description of what they discovered from the data, classifying information into seven themes and providing an interpretation of the findings. Both the researcher and an independent reviewer met after analysing the data independently to agree on the themes.

#### Ethical consideration

In order for researchers to maintain the highest standard of ethical practice, the following steps were taken. Firstly, the researcher engaged the services of librarians at the North-West University for assistance. The promoters perused and made inputs at each phase of the review. The services of an independent reviewer were engaged for the trial run for the first three articles and a consensus was reached, indicating that the review question and methodology were appropriate to achieve the stated objective. In addition, PRISMA flow chart was used, the appraisal tables were presented and sources used in this review were acknowledged in the text and in the list of references.

This article followed all ethical standards for this research without direct contact without human or animal subjects. Ethical approval was obtained from North-West University Health Science Ethics Committee (NWU-HSEC), reference number: NWU-00633-18-A9, 02 October 2018.

### Step 5: Results

The results of this systematic review yielded seven themes, which are as follows: information sharing, empowerment of key role-players, programme development, commitment from key role-players, quality improvement executive support and collaborative partnerships.

## Theme 1: Information sharing

Studies have revealed that information on the dissemination and implementation of a policy on school health should be shared openly and effectively amongst stakeholders without citing any specific evidence (Larsen et al. 2017:40; Larson et al. 2017:208; Montague et al. 2014:3; Tapia et al. 2017:528; Weatherson et al. 2017:835). Other studies have considered feedback, telephone calls, consultations, dialogue and referral as evidences (Leow et al. 2014:108; Nguyen et al. 2017:18; Vanneste et al. 2016:6; Weatherson et al. 2017:83). For instance, dialogue, as a means of sharing information to promote active participation of stakeholders was mentioned by Vanneste et al. (2016:6). Chiang et al. (2015:779) used telephone calls as evidence used by school managers for a given period to identify problems and improve compliance with the school canteen menu. Wolfenden et al. (2017:6) indicated academic detailing as an evidence used to disseminate and implement a policy on school health. In the school, children were randomised to receive 12–14 months multi-strategic evidence. The evidence amongst others improved implementation and support of a school nutritional policy. Other studies have used evidence-based material as proof of information sharing to disseminate and implement a policy on school health (Nathan et al. 2016:106; Robinson et al. 2014:S74) For example, a study revealed improvement of evidence in implementation of school-wide positive behaviour programme by using evidence-based materials (Nathan et al. 2016:106). Another study revealed menu audit reports as impartation evidence to disseminate and implement school health services (Robinson et al. 2014: S76).

## Theme 2: Training and development of key role-players

Studies have revealed that training and development of key role-players should be performed to develop professionals, but specific approaches to be adopted to achieve the desired goal were not stated in these studies (Nathan et al. 2016:106; Reilly et al. 2017:218; Yoong et al. 2016:126). Out of these studies, two of the studies focussed on regular teaching and training of key role-players as evidence (Reilly et al. 2017:218; Yoong et al. 2016:126). Other studies revealed that the development of key role-players should be best done through creation of awareness, knowledge and recruitment of the workforce (Larson et al. 2017b:208; Nguyen et al. 2017:18; Reilly et al. 2017:218; Tapia et al. 2017:530). Another study by Wolfenden et al. (2017:6) revealed a form of motivation to stimulate interest key role-players as another evidence.

## Theme 3: Programme development and research

The review revealed studies that advocated the need for programme development and research as evidence to disseminate and implement policies on school health, whereas other studies revealed measures in which the programme and research materials would be the following: delivery of evidence-based information, development of policies and action guides (Chiang et al. 2015:779; Ha et al. 2018:11; Nathan et al. 2016:106; Robinson et al. 2014:S76). However, other studies have revealed that programme manuals have been adopted, and theories have been tested and used as evidence to disseminate and implement a policy on school health; thus, such evidence should be utilised (Chiang et al. 2015:779; Larson et al. 2017b:208; Tapia et al. 2017:530; Vanneste et al. 2016:6). For instance, Larson et al. (2017:208) focussed specifically on the positive use of RE-AIM framework (an evidence-based framework) to implement and disseminate school health nutritional programmes. The study revealed that roughly 47% of parents who participated in the study observed changes in their children’s positive attitude towards fruits, vegetables and other healthy snacks.

## Theme 4: Commitment from key role-players

Studies have confirmed the commitment of key role-players involved in school health in different ways towards the dissemination and implementation of policies on school health. For instance, studies have revealed the commitment of school children (Ha et al. 2018:11; Pagnotta et al. 2016:297; Pucher et al. 2017:8). An examination of one of the studies revealed that a school-based recruitment programme, voluntary medical male circumcision (VMMC) and a three-phased demand-creation evidence were used to commit school children (Pagnotta et al. 2016:297). Considering the success recorded, it was concluded that demand-creation evidence, when adapted, has the potential to sustain school-based implementation and promotion of a policy on school health (Pagnotta et al. 2016:297). One study revealed the commitment of key role-players involved in school health without stating such key role-players (Pucher et al. 2017:8). Two studies revealed the commitment of teachers (Nathan et al. 2016:106; Yoong et al. 2016:126). Three studies underscored parents’ family and community involvement and/or consultation (Larson et al. 2017:204–213; Montague et al. 2014:3; Pagnotta et al. 2016:297). The findings by Larson et al. (2017:208) substantiated the use of local resources as an appropriate evidence.

## Theme 5: Monitoring activities

This review revealed studies that have highlighted this theme, monitoring activities, as evidence to disseminate and implement a policy on school health. Two of these studies suggest that monitoring activities should be through direct observations and on-site visits, self-report or menu-audit (Flannery et al. 2013:272; Robinson et al. 2014:S76). On the other hand, other studies have revealed regular or periodic reviews, accountability and distribution of questionnaire and impromptu visits. Others used self-assessment and regular feedback to disseminate and implement a policy on school health (Larsen et al. 2017:41; Larson et al. 2017:208; Pagnotta et al. 2016:297; Wolfenden et al. 2017:6; Yoong et al. 2016:126). Out of these, one study was particularly about self-assessment (Larsen et al. 2017:41).

## Theme 6: Executive support

This review established that with regard to executive support, at all levels of school health, key role-players are important in the dissemination and implementation of a policy on school health. As a result of the significant number of studies that identified with this theme, it is regarded as a crucial variable to ensure an effective policy on the dissemination and implementation of school health. For example, it was stated in two studies that effective dissemination and implementation of a policy on school health require the provision of resources and executive support (Flannery et al. 2013:272; Reilly et al. 2017:218). The services, amongst others, adopted in these studies, were ongoing support, provision of resources and executive support, but it was never indicated to what extent these support services could be rendered.

Other studies have revealed that executive support should cover financial and technical materials, as well as leadership and political support (Chiang et al. 2015:779; Hager et al. 2016:746; Nathan et al. 2016:106; Wolfenden et al. 2017:6). Hager et al. (2016:742–50) were very emphatic about financial assistance in the form of grants and in-sort donations. Other studies considered institutional support, system and programme support, external and internal support, leadership support and staff support (Flannery et al. 2013:270; Hager et al. 2016:746; Larsen et al. 2017:40; Montague et al. 2014:3; Tapia et al. 2017:530). The remaining three studies considered development of stakeholders through creation of training opportunities and facilities, supportive teachers’ behaviour and decentralisation of services (Larson et al. 2017:208; Pucher et al. 2017:8; Yoong et al. 2016:126).

## Theme 7: Collaborative partnership

Some studies have identified advocacy and collaboration, partnership, integration and consensus building and formation of advisory boards as evidences for the dissemination and implementation of a policy on school health. Moreover, some studies have explicitly mentioned collaboration and partnership, an indication that these two evidences are widely considered (Flannery et al. 2013:272; Hager et al. 2016:746; Larson et al. 2017:208; Montague et al. 2014:3; Weatherson et al. 2017:835). For instance, in one qualitative study, the authors retroactively examined how three states facilitated creation and administered heat-acclimatisation guidelines in high schools and shared authority and leadership through collaboration (Weatherson et al. 2017:835). Similarly, three studies, by implication, revealed that integration, advocacy and consensus building are evidences, but never provided an explanation, what, how, by whom and with whom to be integrated to arrive at a consensus (Larson et al. 2017:208; Weatherson et al. 2017:835; Yoong et al. 2016:126). One study confirmed development of advisory board as an evidence (Flannery et al. 2013:272).

## Discussion

This systematic review offered existing evidences on the dissemination and implementation of a policy on school health under seven themes. The findings of the systematic review revealed that if these evidences are adopted, a policy on school health would be effectively disseminated and implemented.

These evidences identified in the studies regarding the dissemination and implementation of a policy on school health seem to be varied and extensively researched in some settings, but there is an evident dearth in most settings. Regardless, these systematic findings are in consonance with previous evidence-based findings, demonstrating that a policy on school health in any setting can be effectively disseminated and implemented. Commonalities of all studies observed in this systematic review were that all the 19 studies adopted have several evidences and criterion, thus explaining why a group of authors appeared under more than one theme. For instance, one study appeared prominently in both shared information and also under quality improvement (Larsen et al. 2017:40). Similarly, another study appeared under empowerment of key role-players as an evidence to conduct nutritional policy in elementary schools in South Africa and also featured under executive support as an evidence (Reilly et al. 2017:218). Another example was a study that adopted several evidences to disseminate and implement a policy on school health under several themes (Tapia et al. 2017:530). Thus, the findings of this study suggest the need for stakeholders to adopt multiple evidences and use appropriate medium to disseminate and implement policies on school health to achieve global school health promotion objectives.

Studies have revealed adequate sharing of information as evidence of dissemination and implementation of a policy on school health. Thus, for these evidences to be effective and useful, amongst several others, stakeholders should use audit reports, regular feedback and telephone calls and actual teaching to disseminate and implement policies on school health. The findings revealed that all stakeholders involved in the telephone calls received adequate training that changed the attitude of school managers to intervene effectively in promoting school health services (Chiang et al. 2015:779; Leow et al. 2014:108). Thus, it could be inferred that the use of appropriate language, dialogue and consultations are considered first necessary interactive processes towards sharing information regarding policies on school health to key role-players. Furthermore, any of these processes, when adopted openly and effectively prior to the actual programme, dissemination and implementation of a policy on school health would be successful (Montague et al. 2014:3; Pucher et al. 2017:8).

Given the fact a substantial number of studies in this systematic review revealed training and development of key role-players as evidence for the dissemination and implementation of a policy on school health, it is suggested that both old and new employees be adequately trained and developed. However, there should be different periods allocated for training and development programmes to be conducted for new and old employees. For instance, these findings revealed the need to develop the potentials of new employees by way of pre-service training before taking up the job to enable them acquire the required skills on the one hand, and on the other hand, continuous professional development trainings should be conducted for older staff to improve their competencies periodically through the use of appropriate local resources (Hager et al. 2016:746; Nguyen et al. 2017:18; Pagnotta et al. 2016:297; Reilly et al. 2017:218; Weatherson et al. 2017:835). Thus, if sustainable knowledge on school health policies and awareness creation programmes are designed and adopted, the potentials of different role-players would be developed, and they will be empowered for the effective dissemination and implementation of a policy on school health.

The findings also revealed that empowerment entails recruiting more workforce, where there is insufficient manpower to assist existing staff and motivate all role-players to arouse their interest towards the effective dissemination and implementation of a policy on school health (Larson et al. 2017:208; Wolfenden et al. 2017:6).

With regard to programme development and research, studies revealed that if school health guidelines are developed and research programmes conducted, stakeholders should use such available evidences to disseminate and implement policies on school health to meet the health, physical and academic needs of school children. This is because the findings revealed that policies, research evidences, action guides and programme manuals and theories used in the past to disseminate and implement policies on school health have been found to promote profound positive impact in the promotion of school health care (Larson et al. 2017:208; Tapia et al. 2017:530; Vanneste et al. 2016:6).

With regard to commitment, studies revealed different key role-players involved in school healthcare to be committed towards the dissemination and implementation of a policy on school health. The variability was linked to the context and targeted population in which the policy was disseminated and implemented. The findings of this systematic review suggest the commitment of school children (Nathan et al. 2016:106; Pucher et al. 2017:8; Yoong et al. 2016:126). Others are the commitment of teachers and the commitment of parents and other relatives according to Larson et al. (2017:208). Furthermore, other studies suggest the commitment of the community (Hager et al. 2016:746) and governmental or non-governmental agencies (Pagnotta et al. 2016:297). However, the findings revealed that none of these players acted on their own accord, but through some underlying motivating factors. These factors include the following: fairness, incentives, promotion of plausible ideas and sharing responsibility were necessary to encourage the effective dissemination and implementation of a policy on school health (Pucher et al. 2017:8). Thus, in developing evidences to disseminate and implement policies on school health, motivating factors must be included. It could, therefore, be inferred that if these factors are considered, a policy on school health would be effectively disseminated and implemented in public schools.

Furthermore, the findings also revealed that six of the seven themes identified in this systematic review need effective monitoring activities of on-going dissemination and implementation of a policy and activities on school health. This is because a good number of studies have revealed monitoring activities as indispensable requirements for the dissemination and implementation of a policy on school health, adding that without this, the objectives of the programme might not be achieved (Chiang et al. 2015:779; Robinson et al. 2014:S74). As such, the findings revealed that monitoring activities should be carried out through, amongst others, observations, on-site visits, self-report or audits (Chiang et al. 2015:779; Robinson et al. 2014:S70). If observations, on-site visits, self-report or audits are adequately utilised, it would enable policy makers and other role players involved in school healthcare to identify the level of dissemination and implementation programmes in terms of progress, process and the impact of the programme on the targeted population. Thus, these findings suggest that effective monitoring activities are critical and as such should adopt appropriate tools such as self-report and audit whilst programmes are on-going. Besides these, the findings also suggest that self-report is a form of self-appraisal and key role-players should, in this light, aspire to regularly appraise the performance of stakeholders with regard to the dissemination and implementation of a policy on school health.

Studies have also revealed that the successful dissemination and implementation of a policy on school health need adequate executive support. The executive support should be in the form of financial and technical material as well as leadership or political support. The implication is that if these support services are provided in any context, these could facilitate the creation of an enabling environment for the training of key role-players to improve skills and proficiencies, develop programmes, conduct research and ensure the commitment of key role-players for the effective dissemination and implementation of policies on school health.

Furthermore, studies have particularly revealed partnership and collaboration of all aforementioned school healthcare players to achieve positive dissemination and implementation results (Larson et al. 2017:208; Montague et al. 2014:3; Nathan et al. 2016:106; Tapia et al. 2017:530; Weatherson et al. 2017:835). These findings suggest that if key role-players involved in school healthcare collaborate with other groups to form collaborative partnership of school healthcare, a policy on school health could be effectively disseminated and implemented. Studies have indicated that collaborative partnership has proved to be a necessary means given that its activities recorded huge success such as expanding each member’s horizon through skills and experience (Wolfenden et al. 2017:6; Yoong et al. 2016:126). In other words, every dissemination and implementation programme was dependent essentially on joint action and collaboration with groups that had same purpose and, as such, should be utilised. The collaboration could be between governmental or non-governmental agencies and developmental partners, essentially for funding and prompt identification of areas that need improvement. In addition, these findings also revealed formation of advisory boards (often experts) under this theme. The findings suggest that if advisory boards are formed by various agencies and governmental bodies, it should provide the necessary assistance in coordinating human and material assets as well as give state-of-the-art directives to school healthcare promoters on the dissemination and implementation of a policy on school health. However, the findings in this systematic review revealed that one study adopted the formation of advisory boards to disseminate and implement such policy (Flannery et al. 2013:272), an indication that formation of advisory boards is considered less an evidence.

## Conclusion and recommendations

Several studies have been conducted on implementation of a policy on school health, but few have focussed on the dissemination of school health services, programmes and policies. This shows that there is scarcity of studies that bear both dissemination and implementation holistically, focussing on evidences used by promoters of healthcare in public schools’ on a policy on school health itself. Thus, it is concluded in this study that if multiple evidences are utilised, a policy on school health would be effectively disseminated and implemented. Effective dissemination and implementation would promote physical well-being, improve children’s school attendance, promote their optimal academic performance and promote educational equality between children in public schools and their counterparts in private schools. Further research is needed in the area of dissemination of a policy on school health using other appropriate methodologies in a wider perspective.
